# 

**Published:** 1889-10

**Authors:** 


CONTENTS

Vol. 36 No. 10.

| | PAGE |
|---|---|
| Looking Forward | 217 |
| An Aim at Looking Forward more Wisely | 221 |
| Starved Nerves and Famished Teeth | 224 |
| The Future Life | 225 |
| Told by a Snake Charmer | 226 |
| Staples Won the Bet | 228 |
| Insomnia | 229 |
| Fruit as a food | 230 |
| Modern Diseases | 232 |
| Satisfy Your Appetite | 233 |
| Special | 235 |
| The N. Y. World's Fair | 235 |
| Sleeplessness | 235 |
| Household | 237 |
| Miscellaneous | 238 |
| Book Notices | 239 |
| Over Their Graves | 240 |

INDEX TO ADVERTISEMENTS OF HALF A PAGE AND OVER.

| | PAGE. |
|---|---|
| Revised Premium List | I |
| Celluloid | II |
| Hollow Suppositories | III |
| E. C. Morris & Co. | IV |
| Lactopeptine | V |
| Catarrh | VI |
| Dennin's Certain Cure | VII |
| An Unprecedented Offer | VIII |
| The Herb Cure Co | X |
| Ladies' Home Companion | XI |

